# Inhibition of leukotrienes and their potential role in type 1 diabetes pathogenesis: implications for montelukast as a therapeutic agent: a case report

**DOI:** 10.3389/fcdhc.2024.1494470

**Published:** 2024-12-19

**Authors:** Pavel Fatulla, Ingela Ström, Christine Lingblom, Marcus Lind

**Affiliations:** ^1^ Department of Medicine, NU Hospital Group, Trollhättan and Uddevalla, Sweden; ^2^ Department of Molecular and Clinical Medicine, Institute of Medicine, The Sahlgrenska Academy, University of Gothenburg, Gothenburg, Sweden; ^3^ Department of Infectious Diseases, Institute of Biomedicine, The Sahlgrenska Academy, University of Gothenburg, Gothenburg, Sweden; ^4^ Department of Clinical Microbiology, Sahlgrenska University Hospital, Gothenburg, Region Västra Götaland, Sweden; ^5^ Department of Medicine, Sahlgrenska University Hospital, Gothenburg, Sweden

**Keywords:** type 1 diabetes mellitus, pathogenesis, eosinophils, montelukast, therapeutic agent

## Abstract

**Introduction:**

Type 1 diabetes involves immune-mediated destruction of insulin-producing beta cells, with eosinophils potentially playing a significant role. Recent studies suggest that leukotriene inhibition might influence this process. This case report presents a novel observation of montelukast, a leukotriene receptor antagonist, reducing insulin requirements in a patient with Latent Autoimmune Diabetes in Adults (LADA). A 55-year-old male with LADA experienced substantial reductions in insulin dosage when treated with montelukast for respiratory symptoms. Initially diagnosed with LADA in 2018, the patient had been on insulin therapy. Montelukast therapy, initiated due to respiratory symptoms, led to a 60.5% reduction in insulin requirements which increased upon discontinuation. A subsequent montelukast course resulted in an 87.9% insulin reduction. Although the insulin-lowering effect diminished with continued montelukast use, the patient reported reduced postprandial hyperglycemia. Blood tests indicated stable glucose levels despite reduced insulin doses.

**Conclusions:**

This case suggests that montelukast may reduce insulin needs in type 1 diabetes patients, potentially through its anti-inflammatory effects on eosinophils. These findings highlight the need for further research into montelukast’s role in type 1 diabetes management and its potential to preserve beta-cell function or reduce insulin dependence.

## Introduction

In recent years, interest in understanding the immunological mechanisms underlying type 1 diabetes has increased, in turn leading to a heightened focus on addressing the primary pathology, which involves the immunological destruction of insulin-producing beta cells in the pancreas. Recently, the first treatment for delaying the onset of type 1 diabetes, utilizing the anti-CD3 therapy teplizumab, was approved in the United States. As all T-cells express CD3, teplizumab inhibits activated T-cells that potentially destroy insulin-producing beta cells (1).

Several immunological mechanisms have been proposed to play a crucial role in the development of type 1 diabetes (2). One potentially significant mechanism involves eosinophils, which are a type of white blood cells engaged in a variety of allergic and inflammatory conditions initiated by T-cells (3). While the role of eosinophils in the development of type 1 diabetes is unknown, some data indicate they may be involved in the destruction of pancreatic beta cells, a central event in the development of type 1 diabetes. In a pilot study, higher levels of immature eosinophils and lower levels of suppressive eosinophils were observed in individuals with type 1 diabetes compared with age-matched controls (4). Suppressive eosinophils have been reported to inhibit activated T-cells (3), which are considered pivotal in the initiation of type 1 diabetes (1).

Levels of galectin-10^hi^, a protein crucial for eosinophils to inhibit T-cells, have been observed to be lower in patients with longstanding type 1 diabetes (4). In this study, a significant number of immature eosinophils were found in all type 1 diabetes patients, and a subset of galectin-10^hi^ eosinophils was entirely absent in all type 1 diabetes patients (4). Moreover, heightened levels of CD4+CD8+ T cells and Th17 cells were noted (4). These findings suggest that the presence of eosinophils, known for their potent ability to suppress T-cells, may contribute to T-cells becoming capable of indiscriminately targeting and damaging insulin-producing beta cells (4).

Currently, there are several medications influencing the immune system that may potentially impact the pathogenesis of type 1 diabetes. Montelukast is one well-known immunomodulatory drug used as an adjunctive treatment for asthma that blocks the cysteinyl leukotriene receptor (CysLTR1), one of the primary receptors of leukotrienes. Leukotriene LTC4, LTD4, LTE4, collectively named cysteinyl leukotrienes (CysLTs), are peptide conjugated lipids that are the products of eosinophils, basophils, mast cells, and macrophages (5).

Considering recent findings indicating that eosinophils play a role in the pathogenesis of type 1 diabetes, we present a case that suggests montelukast may hold therapeutic potential in managing the disease. This case demonstrates a possible reduction in insulin requirements in a patient with type 1 diabetes during montelukast treatment, likely related to its anti-inflammatory effects on eosinophils. Further research and clinical trials are necessary to comprehensively assess the therapeutic role of montelukast in type 1 diabetes management.

## Case description

A 55-year-old male diagnosed with latent autoimmune diabetes in adults (LADA) type 1 diabetes in December 2018, with an initial HbA1c of 68 mmol/mol (8.4%) and no familial history of diabetes but a notable genetic predisposition for cardiovascular disease on both maternal and paternal sides.

Following a wrist injury requiring endoscopic intervention, the patient exhibited elevated blood pressure, hypercholesterolemia, and hyperglycemia. He received antihypertensive and cholesterol-lowering medications, along with metformin and dietary adjustments. Minimal improvement in glucose levels prompted suspicion of type 1 diabetes, which was confirmed with anti-GAD levels at 28 IU/mL (<10IU/mL). ICA titer was negative. Treatment began with insulin glargine, 6 units/day in May 2019.

The patient was referred to a specialist while on 7-8 units/day of insulin glargine and 1-2 units of bolus insulin before meals. At that time, he had long-standing asthma-like symptoms and was using Bufomix once daily, Buvnethol three times before exercise, and trialed on Relvar, which did not alleviate his respiratory symptoms. Simultaneously, Conn’s syndrome was diagnosed and managed under endocrinological care.

At the initial diabetes clinic visit in May 2019, the patient demonstrated preserved endogenous insulin production. Insulin glargine was increased to 10 units and accompanied by detailed instructions to regulate the dosage, allowing for a maximum of 15 units as required. Mealtime insulin was discontinued, and HbA1c declined to 44 mmol/mol (6.2%).

Diabetes management evolved from May 2019 to June 2022. Beginning with the introduction of Libre in May 2019, the patient maintained regular physical activity, dosages of insulin glargine and lispro were adjusted, and the patient transitioned to the Omnipod Dash pump in April 2021.

As of July 2021, the patient was utilizing the pump with a basal dose of 8.3 units per day and bolus doses ranging from 10-12 units per day. On January 25, 2022, treatment settings were adjusted to 0.25 units per hour from midnight to 7:30 AM, 0.25 units per hour from 7:30 AM to 7:00 PM, and 0.3 units per hour from 7:00 PM to midnight.

In March 2022, the patient was prescribed montelukast 10 mg orally for upper respiratory symptoms developing one month after fully recovering from COVID-19. The patient had been feeling well during the interim but developed worsening asthma-like symptoms after receiving the second dose of the COVID-19 vaccine. No other treatments such as oral corticosteroids were used during this time. From March until May, the patient used montelukast on two separate occasions each lasting a few weeks and had been without montelukast during two distinct periods. Montelukast use was associated with a noteworthy reduction in the average daily insulin requirement. On April 1, 2022, the patient subjectively noted a decrease in overall dependence on insulin and, importantly, affirmed that he had not modified his dietary patterns or level of physical activity.

The patient believed that montelukast had positive effects in the form of decreased need for insulin during periods of montelukast treatment which was indicated for upper respiratory symptoms. At this time there were also studies indicating that eosinophils could play a role in the pathogenesis of type 1 diabetes, and in November 2022 the patient was continued on montelukast 10 mg daily. Blood samples were collected, and mixed meal tolerance test (MMTT) was performed to assess potential improvement in insulin production.

MMTT was performed before the patient resumed montelukast treatment in November 2022, as well as in December 2022 and March 2023. On each occasion, blood samples were collected, including Islet cell autoantibodies and a complete blood count with differential. Montelukast 10 mg daily was continued until September 2023.

The patient reported full adherence to the intervention with no issues related to tolerability. There were no adverse or unanticipated events noted during the treatment period, as assessed through regular patient consultations.

## Timeline

**Figure 1:**
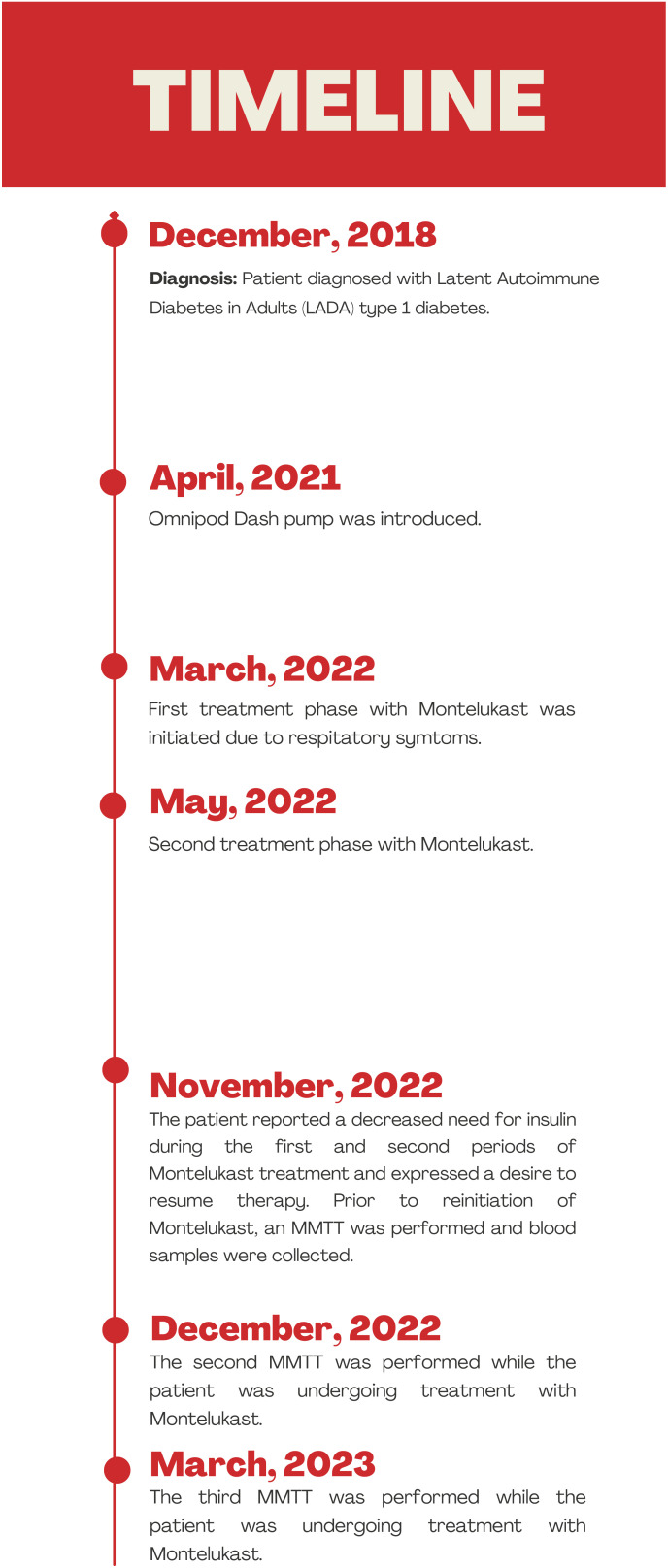
Figure illustrates a timeline of events from the episode of care, including the periods of montelukast treatment and the corresponding MMTT assessments.

**Figure 1** illustrates a timeline of events from the episode of care, including the periods of montelukast treatment and MMTT assessments.

## Diagnostic assessment

**Figure 2:**
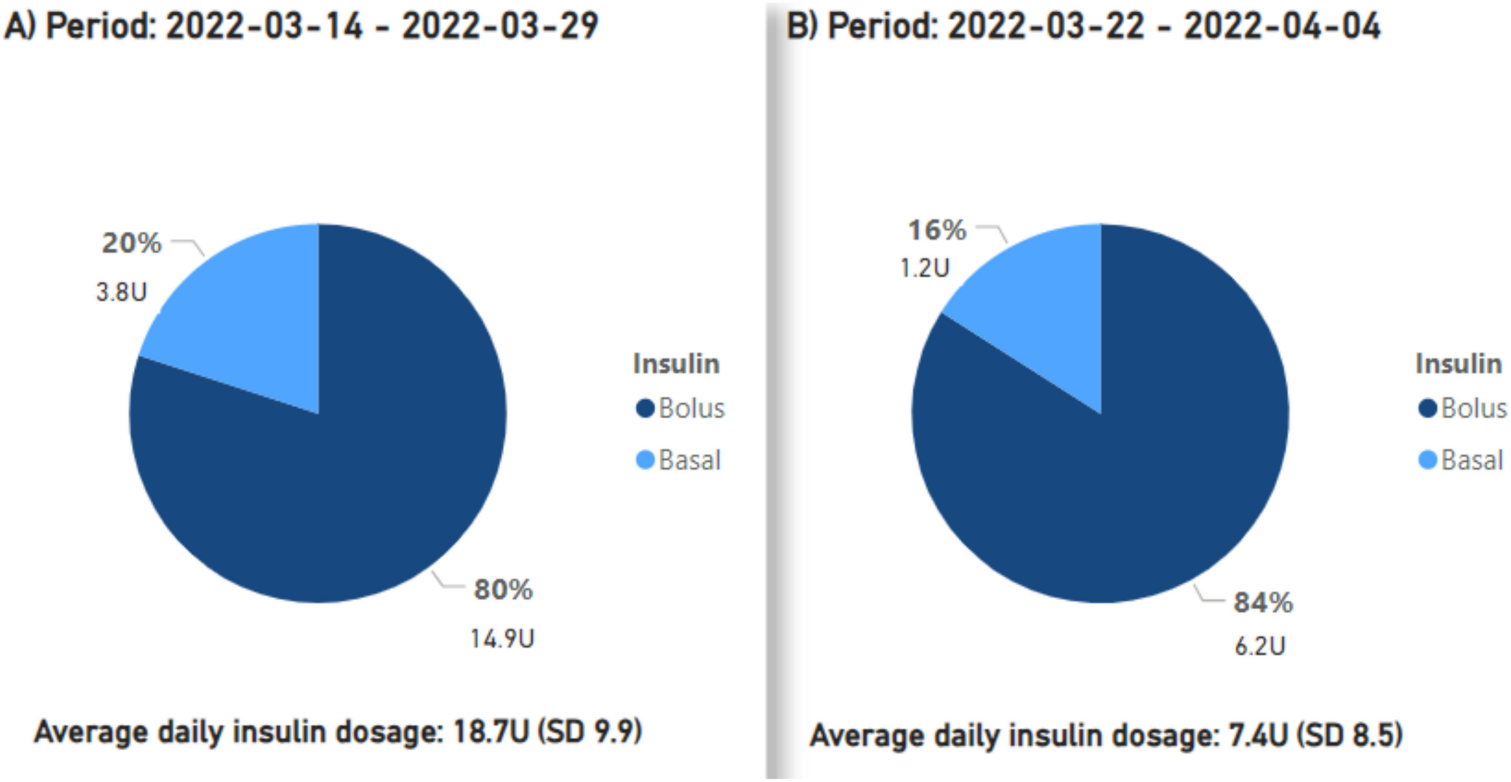
The proportions and cumulative units of basal and bolus insulin requirements are presented, along with the average daily insulin demands. Period **(A)** signifies the pre-initiation phase of treatment with montelukast, period ‘**(B)** corresponds to the first treatment phase with montelukast.

**Figure 3:**
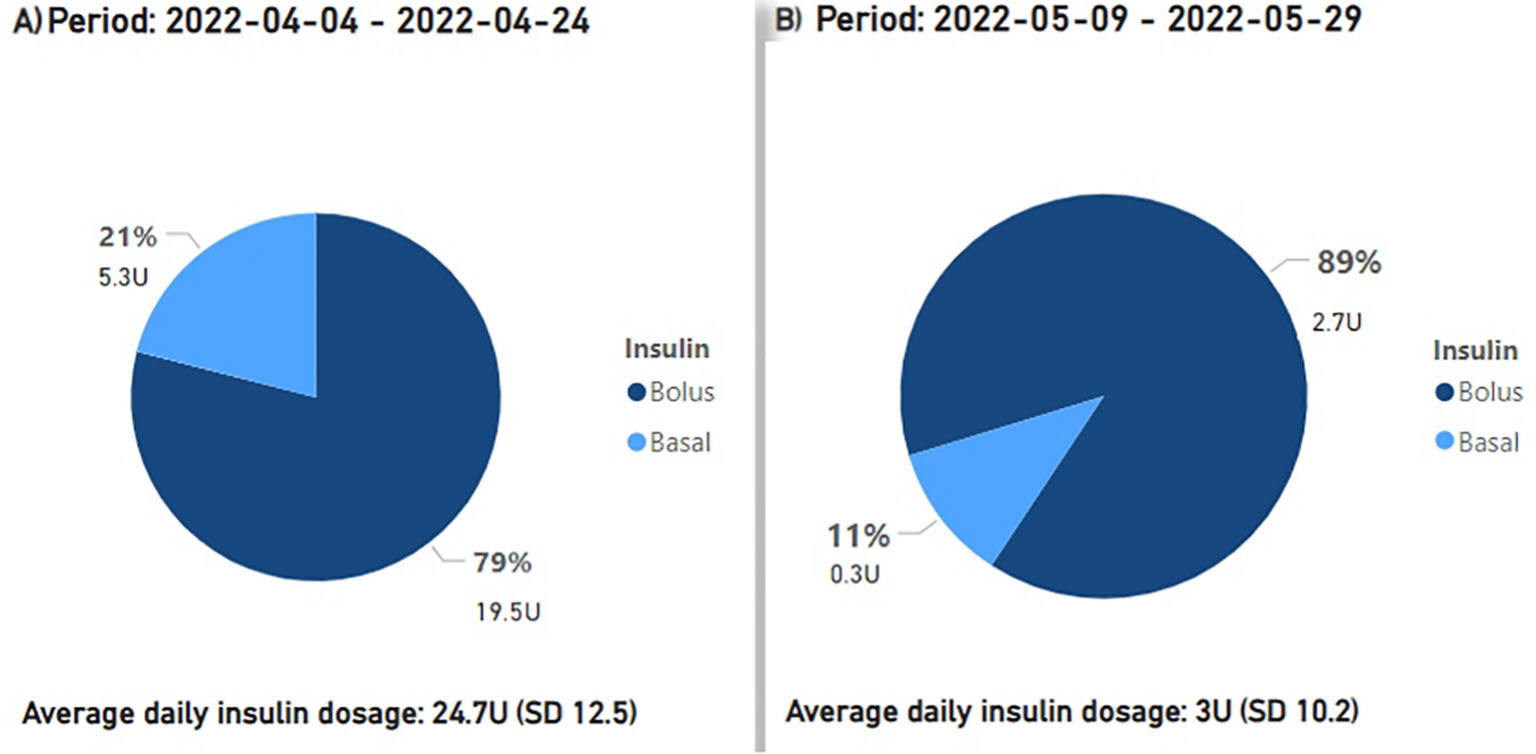
The proportions and cumulative units of basal and bolus insulin requirements are presented, along with the average daily insulin demands. Period **(A)** signifies the period before the second phase of treatment with montelukast, period **(B)** corresponds to the second treatment phase with montelukast.

Preceding the introduction of montelukast from March 14 to 29, 2022, the mean daily insulin dose stood at 18.7 units. From March 22 to April 4, 2022, while employing montelukast therapy, the mean daily insulin dose declined to 7.4 units, or a 60.5% reduction (**Figure 2**). After montelukast was discontinued, the mean daily insulin dose increased to 24.7 units during the period April 4-24, 2022. During a subsequent course of treatment with montelukast in May 2022, there was an additional reduction in the mean daily insulin dosage to 3 units, signifying an 87.9% decrease from the prior month (**Figure 3**), but an increase was observed once again when treatment was discontinued.

Continuous glucose monitoring (CGM) during the two periods of montelukast use and two periods of non-use revealed no significant difference in mean glucose levels, with values ranging between 6.6-6.9 mmol/L (118.8-124.2 mg/dL) throughout. Glucose levels remained consistently stable, including mean standard deviations ranging from 1.6-1.7mmol/L (28.8-30.6 mg/dL). Time spent within the target range of 4.0-9.0 mmol/L (72.0-162.0 mg/dL) varied between a minimum of 86% and a maximum of 91%. The percentage of high blood glucose values ranged from 9-12%, while low values were observed at 1-2%, thus indicating decreased insulin needs with glucose levels remaining similar overall.

When the patient resumed montelukast in November 2022, similar insulin dose reductions were not observed. The patient also reported that he could not reduce insulin doses to the same extent as when montelukast was initiated earlier in 2022 but did report that hyperglycemia after meals became less pronounced. In November 2022, the patient exhibited a leukocyte count totaling 5.7x10^9/L, with eosinophils accounting for 0.17x10^9/L. Following treatment with montelukast in December, the leukocyte count was measured at 5.6x10^9/L, with eosinophils showing an increase to 0.21x10^9/L. By March 2023, the leukocyte count had risen to 6.3x10^9/L, with eosinophils comprising 0.22x10^9/L of the total count. In the MMTT conducted before the patient resumed treatment with montelukast in November 2022 and subsequently in December 2022 and March 2023, we did not observe any clear change in C-peptide levels compared to levels before treatment. Area under the curve for C-peptide (AUC CP) from the MMTT was 0.90 nmol/L in November 2022 and 0.71 nmol/L and 0,76. nmol/L in December 2022 and March 2023, respectively. BMI ranged between 27.2 to 28.1 kg/m2 during the follow-up period.

## Discussion

Our observations of montelukast treatment in a patient with LADA can be summarized as follows. Initially, the patient experienced a significant decrease in insulin requirements during two separate periods while on montelukast, despite maintaining consistent dietary habits and levels of physical activity. Simultaneously, blood glucose levels remained stable as insulin needs decreased. However, in the third round of montelukast treatment we did not observe a similar decline in insulin doses. Nonetheless, the patient himself reported a reduction in post-meal hyperglycemia.

It is difficult to explain precisely why the patient was able to reduce insulin doses during the first two episodes of treatment with montelukast but not later on. Notably, the patient was not on other medications such as glucocorticoids at the beginning of treatment or thereafter that could have influenced outcomes, nor were there any changes in lifestyle factors such as diet or exercise. We observed no improvement in the MMTT; however, it is interesting that the patient reported subjective benefits. Specifically, when montelukast was discontinued, blood glucose spikes were more pronounced when bolus insulin doses were missed compared to when montelukast was used and bolus doses were missed. During montelukast treatment, the patient observed less significant glucose spikes even with lapses in bolus insulin administration. Unlike the first and second rounds of montelukast treatment, insulin doses were not reduced thereafter making MMTT comparisons difficult and a limitation of this case report as MMTT was not performed at the outset of treatment, although at the time the patient was not aware of the potential impact montelukast would have on insulin requirements.

These findings suggest that treatment with montelukast had beneficial effects in this patient with LADA diabetes. Furthermore, these observations are noteworthy considering our current understanding of the immune system, characterized by a multitude of cells and cytokines working in collaboration to regulate immune responses. Consequently, different mechanisms may contribute to the development of immunologic destruction of pancreatic beta cells, presenting potential targets for delaying or enhancing insulin production in type 1 diabetes.

Individuals genetically predisposed to type 1 diabetes present with a dynamic onset of the illness characterized by distinct stages. The initial stage is marked by the presence of autoantibodies, followed by a phase of dysglycemia, and ultimately an impaired response to glucose load, all stages where the need for exogenous insulin is absent (1). The progression provides critical junctures for strategic intervention. The ability to diagnose the condition prior to clinical onset signifies a substantial advancement in the field. This knowledge was pivotal when the first immunological treatment targeting CD3 for preventing the onset of type 1 diabetes was approved in the USA (1). In our specific case, it is noteworthy that the patient initially demonstrated a positive response to montelukast, resulting in reduced insulin requirements. However, this response diminished over time while the patient still experienced fewer postprandial hyperglycemic episodes. This emphasizes the potential advantages of customized interventions at different stages, especially in situations where a degree of preserved endogenous insulin production exists, and indicates that if montelukast is administered at the onset of beta cell destruction the outcome may be more favorable.

Given the findings of a previous pilot study which identified higher levels of immature eosinophils and lower levels of suppressive eosinophils in individuals with type 1 diabetes (4), the potential impact of montelukast as an anti-inflammatory agent warrants special consideration. It is plausible that individuals with type 1 diabetes exhibiting impaired eosinophils may benefit therapeutically from montelukast. Its mechanism of action involves acting as a CysLT1 antagonist, effectively impeding the binding of the potent inflammatory agents cysteinyl leukotrienes to their receptor sites on eosinophils. This inhibition may be particularly relevant in cases where eosinophil function is impaired, especially given their known tendency towards an anti-inflammatory role. Today, there is increased understanding of eosinophils and their anti-inflammatory role (3). Disrupted eosinophils may possess diminished anti-inflammatory capabilities due to their immaturity. Montelukast may restore impaired eosinophilic anti-inflammatory function, subsequently influencing other immune cells through distinct signaling pathways leading to less immune-driven inflammation in the pancreas, potentially contributing to heightened insulin production and reduced insulin dependency, as observed initially when the patient commenced montelukast.

In this case, our proposed mechanism of action suggests that montelukast inhibits the function of inflammatory and/or immature eosinophils while concurrently promoting the presence of suppressive eosinophils. This aligns with earlier research confirming a notable deficiency in suppressive eosinophils among individuals with type 1 diabetes compared to their healthy counterparts (4). Suppressive eosinophils hinder activated T-cells by utilizing the intracellular protein galectin-10 (3). Diminished levels of suppressive eosinophils may potentially allow activated T-cells to target and damage the insulin-producing beta cells.

It is important to recognize that this case report is based on a patient with LADA, which may pose challenges to fully draw parallels to individuals with type 1 diabetes at younger ages. While both conditions share a pathogenic immunologic mechanism that leads to the destruction of pancreatic beta cells with many similarities including elevated islet cell autoantibodies, they can exhibit clinical differences in their presentation. Future prospective studies should consider including patients with type 1 diabetes separately from those with LADA to enhance our understanding of the unique characteristics and treatment responses associated with each condition.

It is also important to recognize the numerous limitations associated with case reports, including their lack of generalizability, inability to establish cause and effect relationships, and the risk of overinterpretation in individual cases. Additionally, individual responses to medications can vary significantly, and the absence of control groups further complicates the interpretation of findings. Nonetheless, we believe that these observations are intriguing and may contribute to further exploration in this area. To date, no studies have investigated this potential immunological approach to restore and possibly enhance insulin production. This case highlights a promising opportunity for future research in the field.

## Patient perspective

The patient is firmly convinced that montelukast contributed to a reduction in insulin doses during the initial treatment periods. Although he was surprised that the same level of reduction did not occur in later stages, he remains firm in his belief that when taking montelukast he experienced fewer peaks in his blood sugar levels.

## Data Availability

The original contributions presented in the study are included in the article/Supplementary Material. Further inquiries can be directed to the corresponding author.
